# Linking Changes in Epithelial Morphogenesis to Cancer Mutations Using Computational Modeling

**DOI:** 10.1371/journal.pcbi.1000900

**Published:** 2010-08-26

**Authors:** Katarzyna A. Rejniak, Shizhen E. Wang, Nicole S. Bryce, Hang Chang, Bahram Parvin, Jerome Jourquin, Lourdes Estrada, Joe W. Gray, Carlos L. Arteaga, Alissa M. Weaver, Vito Quaranta, Alexander R. A. Anderson

**Affiliations:** 1 Integrated Mathematical Oncology, H. Lee Moffitt Cancer Center & Research Institute, Tampa, Florida, United States of America; 2 Department of Oncologic Sciences, University of South Florida College of Medicine, Tampa, Florida, United States of America; 3 Division of Tumor Cell Biology, Beckman Research Institute of City of Hope, Duarte, California, United States of America; 4 Vanderbilt Integrative Cancer Biology Center, Vanderbilt University Medical Center, Nashville, Tennessee, United States of America; 5 Life Sciences Division, Lawrence Berkeley National Laboratory, Berkeley, California, United States of America; University of California San Diego, United States of America

## Abstract

Most tumors arise from epithelial tissues, such as mammary glands and lobules, and their initiation is associated with the disruption of a finely defined epithelial architecture. Progression from intraductal to invasive tumors is related to genetic mutations that occur at a subcellular level but manifest themselves as functional and morphological changes at the cellular and tissue scales, respectively. Elevated proliferation and loss of epithelial polarization are the two most noticeable changes in cell phenotypes during this process. As a result, many three-dimensional cultures of tumorigenic clones show highly aberrant morphologies when compared to regular epithelial monolayers enclosing the hollow lumen (acini). In order to shed light on phenotypic changes associated with tumor cells, we applied the bio-mechanical *IBCell* model of normal epithelial morphogenesis quantitatively matched to data acquired from the non-tumorigenic human mammary cell line, *MCF10A*. We then used a high-throughput simulation study to reveal how modifications in model parameters influence changes in the simulated architecture. Three parameters have been considered in our study, which define cell sensitivity to proliferative, apoptotic and cell-ECM adhesive cues. By mapping experimental morphologies of four *MCF10A*-derived cell lines carrying different oncogenic mutations onto the model parameter space, we identified changes in cellular processes potentially underlying structural modifications of these mutants. As a case study, we focused on *MCF10A* cells expressing an oncogenic mutant *HER2-YVMA* to quantitatively assess changes in cell doubling time, cell apoptotic rate, and cell sensitivity to ECM accumulation when compared to the parental non-tumorigenic cell line. By mapping *in vitro* mutant morphologies onto *in silico* ones we have generated a means of linking the morphological and molecular scales via computational modeling. Thus, *IBCell* in combination with 3D acini cultures can form a computational/experimental platform for suggesting the relationship between the histopathology of neoplastic lesions and their underlying molecular defects.

## Author Summary

**Figure 1:**
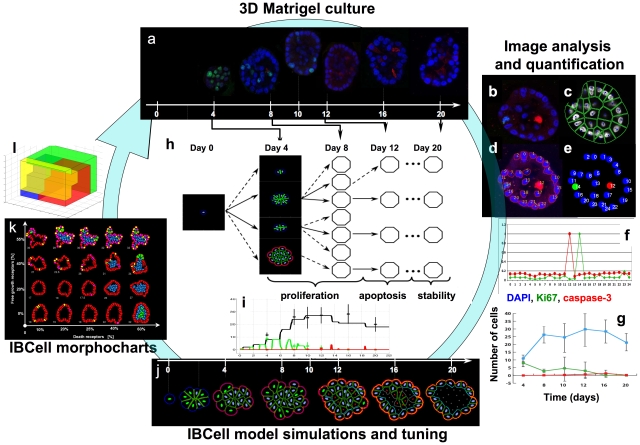
A quantitative integrative approach to model the development of normal acini and their mutants. (a) 3D Matrigel culture of non-tumorigenic epithelial breast cell line *MCF10A*. The representative data at days: 4, 8, 10, 12 16 and 20 show central cross sections through the developing acini stained for cell nuclei (DAPI, blue), cell proliferation (Ki67, green) and cell apoptosis (caspase-3, red). Quantitative image analysis: (b) confocal microscopy images stained for DAPI, Ki67 and caspase-3 are (c) segmented using the *BioSig* software and (d) used to delineate cell nuclei. *BioSig* is used to identify the intensities (f) of red and green wavelengths in each nuclei that allows for reconstruction (e) of proliferative and apoptotic events in the stained acinus and for determination of counts of growing (green), dying (red) and the total number (blue) of cells during the whole course of the experiment (g). The computational *IBCell* model of acinar morphogenesis has been tuned with *MCF10A* data by constructing the search tree (h) that identifies several model parameters (tree branches) generating the desired structures and cell counts on the consecutive time points. (i) The evolution of growing (green), dying (red) and the total number of cells (black) arising during the *MCF10A*-tuned simulation; stars represent the average value from experimental data. (j) The simulated morphologies at the days corresponding to experimental data; nuclear staining: viable cells (blue), growing (green), dying (red); membrane receptor staining: adhesive receptors (green), growth sensors (blue), ECM receptors (pink-red-orange depending on ECM concentration), apical markers (cyan), death receptors (grey). (k) 2D morphocharts showing a collection of final acinar morphologies arising for different combinations of growth and death sensors thresholds; (l) 3D morphochart parameter space representing combinations of growth, death and ECM sensor thresholds that result in normal (red region), not-hollow (blue), degenerate (yellow) and non-stabilized (green) acini.

The majority of tumors arise in epithelial tissues that form monolayers of tightly packed cells enclosing the inner ductal or lobular cavities. Epithelial tumors (carcinomas) are associated with a disruption of epithelial architecture, such as filling of the inner lumen in the early stages of cancer, or the distortion of the ductal structure and spreading to the surrounding stroma in the subsequent invasive stages of tumor. Non-tumorigenic epithelial cells grown in 3D *in vitro* cultures form regular monolayered spheroids with hollow lumen (acini, Fig. 1a) resembling the architecture of normal epithelial cysts. In contrast, tumor cells taken from patients' biopsies and grown in 3D culture acquire various morphologies, often loosing the epithelial-like architecture. How these molecular defects produce such abnormal morphologies remains an open issue. We propose here to use the bio-mechanical model of epithelial morphogenesis, *IBCell*, to quantitatively investigate the phenotypical changes that the epithelial cells need to obtain in order to produce the aberrant morphologies observable experimentally and clinically. *IBCell* in combination with 3D acini cultures can form a computational/experimental platform for suggesting the link between histopathology of early tumors and underlying molecular defects.

## Introduction

The environment in which tumor cells are growing *in vivo* can be very complex, and may include distinct stromal cells, normal or aberrant vasculature, inhomogeneous concentrations of nutrients, proteases or growth factors, gradients in interstitial pressure or non-uniform alignment and cross-linking of various fibrous proteins forming the extracellular matrix (ECM). Since the cells are exposed to these various and often contradictory microenvironmental cues, and moreover, they can actively participate in remodeling of the physical structure and chemical composition of the stroma, it is difficult to predict tumor progression and response to treatments. The change in cell phenotypic state (*i.e.*, the initiation of cell proliferation or death, cell epithelial polarization or epithelial-mesenchymal transition) depends not only on cell intrinsic sensitivity to extrinsic cues from the surrounding microenvironment, but also on cell robustness and adaptability to microenvironmental conditions.

Several *in vivo* techniques have been used to investigate interactions between individual cells and to test cell responses to various extrinsic cues in more controlled conditions. In particular in the three-dimensional (3D) culture systems cells display many features characteristic of their *in vivo* growth, but not observed when these cells are cultured in two-dimensional monolayers. Ideally, one would like to be able to make an initial assessment about the possible molecular changes or underlying mutations by examining the morphology of the multicellular structures grown from mutated or tumorigenic cells.

Therefore, we have developed a computational model, *IBCell* (Immersed Boundary model of a Cell [1], [2]) that allows us to simulate the development of multicellular structures by focusing on cell mechano-biology and the interactions between individual cells and their microenvironment. *IBCell* is a general computational framework that has been previously used to model different tumor related phenomena, such as growing multiclonal colonies [3], various patterns of ductal carcinoma in situ [4], and formation of invasive cell cohorts [5]. The advantage of the *IBCell* model over other cell-based modeling approaches in which cells are represented either as point particles or as deformable cells composed of fixed size grid sites [6], [7], [8], [9] lies in the fact that the cells in our model are fully deformable. Cell geometry in *IBCell* is neither predefined nor grid-determined, but can vary dynamically due to interactions between individual cells. Moreover, the plasticity of cell shape is accompanied by dynamical changes in cell sensors/receptors configuration. Thus two neighboring cells or two phenotypically identical cells may have slightly different distributions of specific cell surface receptors leading to a natural cell-to-cell heterogeneity, which is similar to what actually happens in real cells. Therefore, *IBCell* model simulations represent more faithfully the emerging multicellular morphologies (such as multilayered structures [2], [10], epithelial acini [11], [12], or ductal carcinoma in situ [4]) than other computational models in which cells are modeled as points, squares, hexagons or spheres [6], [8], [9], [13], [14]. This makes the outcomes of *IBCell* simulations more easily comparable with experimental morphologies in both qualitative (shape) and quantitative (cell numbers) ways.

The rest of this paper is organized as follows. We first summarize how *IBCell* has been adjusted to model the formation of epithelial acini by focusing on three cellular processes: cell proliferation, cell apoptotic death and ECM-density dependent inhibition of cell growth. Then we show how the model can be tuned, so the emerging acinar structures match qualitatively and quantitatively the experimental data collected from 3D culture of a non-tumorigenic breast cell line *MCF10A* [15]. We then use this *MCF10A*-tuned model to explore the 3D parameter subspace (the *IBCell* morphochart) corresponding to different non-stabilizing acinar mutants and compare these simulated structures with the experimentally observed morphologies of *MCF10A-HER2* mutants. In addition, we analyze more closely the *IBCell* simulation reproducing the *MCF10A-HER2-YVMA* [16] mutant morphology to quantitatively assess the changes in model parameters when compared to the simulation reproducing the wild-type *MCF10A* acinus. This allows us to formulate hypotheses about the phenotypic changes that have occurred in the mutated cells and to potentially guide further experimentation.

## Results

### *IBCell* model of epithelial acini: assumptions and challenges

The generic *IBCell* model has been adjusted to represent interactions between individual cells that collectively lead to the formation of hollow multicellular structures (acini) composed of a shell of epithelial cells enclosing a hollow lumen. As our experimental prototype, we used human epithelial breast cells, *MCF10A*, and followed their development in a 3D Matrigel culture as described in [15], [17], [18]. In particular, our goal was to capture several stages of MCF10A acini formation starting from a single cell and including self-organization into a multicellular cluster, emergence of inner and outer cell subpopulations, epithelial polarization of all outer cells as well as death of the inner cells and lumen formation. An important feature of this model is the use of cell surface receptors/sensors to define cell interactions and communication with the surrounding environment. The following five kinds of receptors/sensors are considered here: (1) cell-ECM receptors that are activated if the concentration of ECM in their vicinity exceeds a prescribed density threshold (similarly to actions of integrins [19]); (2) cell-cell adhesive receptors that define an adhesive link between two distinct cells located sufficiently close to one another (similarly to cadherins [20]); (3) cell apical markers [21] that emerge in an outer polarized cell by disassembling all existing cell-cell adhesive links with inner cells; (4) cell death receptors [22] that are created in an inner cell upon its detachment from the polarized cell or from another dying cell; (5) cell growth sensors that are used by the cell to sense free space in their vicinity that is necessary for the initiation and progression of cell growth – this is the assumed default state of all sensors when they are not engaged in the other processes listed above.

The phenotypic state of each cell, *i.e.*, its growth, death, senescence or epithelial polarization, is modulated by the percentage of receptors/sensors recruited to a particular process. For example, the host cell can grow only if it can sense sufficient space in its vicinity, which is defined by a percentage of growth sensors located on its surface. If this ratio is small because of an excess of cell-cell or cell-ECM adhesion receptors, the host cell is considered to be in contact inhibition. Similarly, the process of cell apoptotic death is initiated when the percentage of cell death receptors reaches a prescribed level. Thus by varying these sensor/receptor thresholds we can specify whether the cell is more sensitive or more resistant to a specific life process, such as cell death, growth, attachment to the ECM or contact inhibition.

Certain assumptions of *IBCell* have proven to be necessary to generate the hollow monolayered structures [11], [12], but may be challenged experimentally in order to falsify model predictions. We assumed that in the developing acinar structures the orientation of dividing cells need to be quite tightly controlled. In our model, all outer cells divide orthogonal to their basal membrane domains (a symmetric division producing two basal daughter cells) in the initial stage of acinar formation, but the mode of cell division is switched in the later stages to asymmetric division that results in emergence of one basal and one luminal daughter cell. This assumption could be verified by systematic inspection of growing cells within the multicellular structure (for instance by using the live-cell confocal microscopy) in order to determine the axes of cell division at the various stages of acinar development. We have previously shown in [12] that improper cell divisions may lead to irregular, degenerate morphologies. We also assumed that stabilization of acinar structures is due to secretion and accumulation of ECM proteins along the membranes of all outer cells, which eventually leads to their growth arrest. Experimentally, one could either inhibit secretion of ECM proteins (*i.e.*, laminin, collagen, elastin and/or fibronectin), or modify cell ECM receptors (*i.e.*, integrins) response, and investigate whether the *MCF10A* cells will still form stable growth arrested structures. We discuss later in this paper how the changes in cell response to ECM cues leads to the emergence of invasive-like mutants. More details about the *IBCell* model can be found in [11], [12]. See also the Methods section for model equations.

### Quantitative modeling of epithelial acini

**Figure 2:**
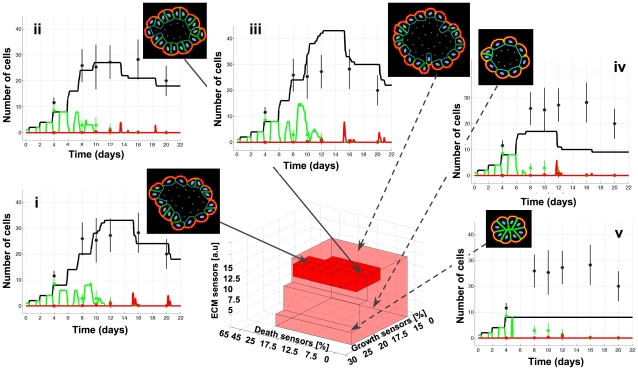
3D *IBCell* morphocharts of epithelial acini. A 3D parameter space of the *IBCell* model defining cell sensitivity to proliferating, apoptotic and ECM-density cues. A light-red region corresponds to the subspace of model parameters that produce hollow acinar structures of various sizes and cell counts (i–v). A dark-red region defines a subspace of model parameters producing acini that quantitatively agree with experimental data from *MCF10A* samples. Two representative final morphologies and evolution curves of cell counts for the *MCF10A*-tuned model are shown in (i) and (ii). (iii) shows an acinar structure larger than experimentally observed *MCF10A* morphologies; the corresponding evolution curve reaches much larger number of cells than the experimental data. (iv) and (v) show much smaller final acini with evolution curves not reaching the level of experimental data. The solid lines represent simulated evolution curves of growing (green), dying (red) and the total number of cells (black); stars represent the average value from experimental data with vertical lines representing the standard deviation.

**Figure 3:**
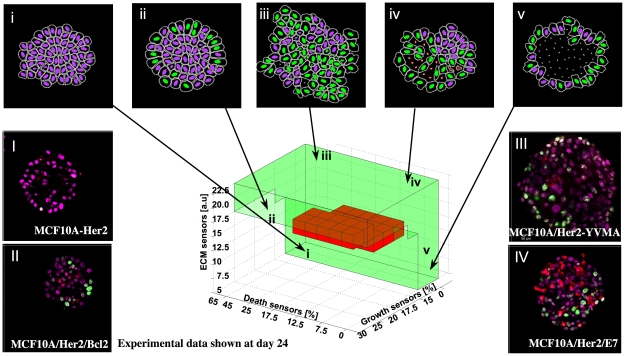
3D *MCF10A*-tuned morphocharts of epithelial mutants. A 3D parameter space of the *MCF10A*-tuned model defining cell sensitivity to proliferating, apoptotic and ECM-density cues. A dark-red region represents a subspace of *MCF10A*-comparable acini. The green region defines a subspace of model parameters producing non-stabilized morphologies representing the potentially invasive mutations of the *MCF10A* baseline: (i) non-polarized structures showing no cell growth; (ii) non-stabilized structures with growing cells observed only on the outer rim; (iii) disorganized structures with multiple cells growing throughout the cell cluster, and with sporadic apoptotic events; (iv) non-stabilized structures with multiple proliferating and apoptotic events; (v) non-polarized but hollow structures with an outer rim containing multiple growing cells. Four genetic mutants of the *MCF10A* parental cell line shown in (I–IV) can be mapped onto the *IBCell* morphochart in a qualitative way by comparing morphologies of simulated (i–v) and experimental (I–IV) cell clusters.

We used an integrative approach (Fig. 1) combining *in vitro* experiments, confocal image analysis and quantification, and high throughput simulation studies to better understand the phenotypic and molecular changes in mutated cells in comparison to their parental non-tumorigenic cell line, *MCF10A*. The multicellular acini grown in the 3D Matrigel culture were collected every four days for 20 days and stained with nuclear marker, DAPI, and antibodies against cleaved caspase-3 and Ki-67, for apoptosis and proliferation, respectively (Fig. 1a). The confocal images of central acinar cross sections (Fig. 1b) were segmented (Fig. 1c) and cellular nuclei delineated (Fig. 1d) using the *BioSig* bioinformatics software (see Methods). Intensities of red (for caspase-3) and green (for Ki67) wavelengths (Fig. 1f) were determined in *BioSig*, and the proliferative and apoptotic events were reconstructed (Fig. 1e) in all considered samples. This allowed for determination of the counts of growing, dying and the total number of cells in all collected samples (Fig. 1g). The previously developed model of epithelial morphogenesis, *IBCell* [11], [12], has been used to simulate the formation of a generic epithelial acinar structure (as described in [11]), and subsequently tuned with experimental data for *MCF10A*. The tuning process requires construction of the search tree (Fig. 1d) that discriminates between model parameters showing promise to generate the desired structure as well as cell counts comparable with experimental data at each considered time point (see Methods) and those that lead to false multicellular structures (see Methods for a description and Fig.S1 for examples). The resulting simulated morphologies from the *MCF10A*-tuned *IBCell* model together with the evolution of growing, dying and the total number of cells matching the experimental data are shown in Figs. 1i–1j. By systematically varying model parameters in *IBCell* simulations we can obtain a spectrum of different final acinar morphologies (a morphochart). A typical chart constructed for parameters defining cell sensitivity to proliferating and apoptotic cues is shown in Fig. 1k. In this morphochart the normal hollow acini are produced by cells resistant to contact inhibition, but sensitive to apoptotic cues (lower left region); partially or fully filled acini arise from cells that are resistant to death signals (right region); and irregularly shaped acini emerge from cells that enter a growth arrest phase due to their sensitivity to contact inhibition (top region). By varying multiple model parameters one can create a multidimensional space of final acinar morphologies and group them according to their architecture. Fig. 1l shows four color-coded regions containing normal (red), filled (blue), irregular (yellow) and non-stabilized (green) acinar structures generated by *IBCell*. For more details on the subspace of normal and non-stabilized acini see Fig. 2 and Fig. 3, respectively.

### 3D *IBCell* morphocharts of epithelial acini

We have previously shown [11], [12] that our *IBCell* model can simulate the development of normal acini starting from a single cell and ending with a structure composed of one layer of tightly packed epithelial cells enclosing the hollow lumen. However, our aim here is not only at qualitative (morphological) agreement with experimental data, but we also want to quantitatively recapitulate the dynamics of the emergent structures. Therefore, the generic model of epithelial morphogenesis has been tuned (as described in Methods) with quantitative experimental data from 3D cultures of *MCF10A* cells. For the tuning process we used counts of proliferating and apoptotic cells, together with information on the times and locations of these events (*i.e.*, initially the proliferating cells are detectable in the whole cluster; at the later stages the growing cells are mostly confined to the outer layer; in contrast the dying cells are only located inside the cluster). The third model parameter that we used, corresponds to the ECM density and may be adjusted to fit experimental data on the density of ECM proteins, such as collagen, elastin, fibronectin or laminin. As a result of this tuning process we identified a set of model parameters that reproduced an acinar morphology in good quantitative agreement with the experimental cellular baseline (Fig. 1i–1j and video S1). This combination of model parameters was then utilized as the initial seed for a suite of simulations that examined the outcome of varying all three thresholds systematically to inspect the whole 3D parameter space (Fig. 2). A broad region in this parameter space can be identified (indicated by light red color) that comprise only of hollow acini of various areas, cell counts and luminal sizes (inserts in Fig. 2i–2v and Fig.S2). A smaller subregion (indicated by dark red color) corresponds to acini that agree quantitatively with the experimental data from the *MCF10A* cell line, *i.e.*, the counts of viable, proliferating and apoptotic cells. Interestingly, the tuned region highlights that there is a degree of variability in the cellular traits and appears to contain two distinct acinar morphologies with slightly different cell counts and evolution curves that both fall within the ranges of *MCF10A* experimental measurements (Fig. 2i–2ii). The area outside the indicated regions contains multicellular morphologies that do not represent normal acinar structures, *i.e.*, they are either distorted, or not hollow, or multilayered and not stabilized (yellow, blue and green regions in Fig. 1l, respectively; see also Fig. 3 and [11], [12], [23] for other examples).

The light-red region specifies the range of acinar plasticity defined here as morphological variations in acinar structure arising as a result of developmental dynamics. Several epithelial systems have been successfully grown in 3D cultures showing variations in acinar sizes both within and across different cells lines (*i.e.*, averaged diameters of prostate acini can reach: 140.8±31.0µm, and 149.8±24.3µm [24], canine kidney acini: 80–90µm [21], breast acini: 67.2±16.5µm and 93.9±19.5µm [15]). In our model such differences in acinar sizes, shapes and cell counts depend on the time at which the acinar structures become growth arrested and stabilized. We hypothesized that extracellular matrix produced by cells acts as an inhibitory mechanism on proliferation. That is, as the density of the matrix increases in the cell vicinity the sensors in contact with the ECM are converted to ECM sensors, thus decreasing the number of growth sensors and inhibiting cell growth. This could be considered biologically equivalent to the process of “matrix assembly”. Therefore, for low values of the ECM threshold the acini stabilize very early reaching only a few cells with a minimal inner lumen (Fig. 2v). With the increasing ECM threshold the acini grow larger and need more time and a higher density of accumulated ECM to become stabilized (compare inserts in Fig. 2iv and Fig. 2iii). Biologically, contact inhibition resembles this kind of behavior, and it is generally observed in 2D tissue culture (*i.e.*, confluence). In a 3D setting, contact inhibition is likely to be more closely related to epithelial polarization, whereby plasma membranes become committed to basolateral and apical domains. Our model recapitulates this mechanism, however, further experiments are needed to confirm which ECM proteins may play a major role in this inhibitory mechanism for a given cell line.

The dark-red region defines robustness of *MCF10A*-comparable acinar structures, *i.e.*, their capability to retain the architecture of the hollow one-layered acini, and to remain in quantitative agreement with experimental data when cell sensors thresholds are varied. Interestingly, the model is less sensitive to death signals, with changes in the death threshold from 7.5% to 17.5% of all cell membrane sensors still resulting in a completely hollow luminal space. Similar effects have been observed experimentally when anti-apoptotic proteins *Bcl-2* or *Bcl-XL* were overexpressed in the *MCF10A* cells [17] causing cells to become more resistant to the initiation of the apoptotic process, but still resulting in formation of the hollow lumen. There is no quantitative data available to assess the relative differences between wild-type *MCF10A* cells and *MCF10A-Bcl-2* or *MCF10A-Bcl-XL* cells in terms of their sensitivity to apoptotic cues, but these experiments show a trend similar to that seen in our simulations. The dark-red region of *MCF10A*-comparable acini in Fig. 2 contains two slightly different morphologies and corresponding evolution curves, both falling within the ranges of experimental measurements. These morphologies are robust with respect to slight variations in the cell proliferation threshold (changes of 15–20%, and 22.5–25% of all cell membrane sensors for ECM threshold of 12.5 and 15, respectively). Again, such variability in acinar sizes, shapes and cell counts is observed experimentally for *MCF10A* cells (Fig. 1g and [15]).

### 3D *MCF10A*-tuned morphocharts of acinar mutants

By tuning the generic model with the experimental data we identified parameter vectors for which the model generates *MCF10A*-like structures. However, when the model parameters are chosen outside this range, the resulting structures are morphologically different from hollowed and monolayered acini. By inspecting the whole *MCF10A*-tuned morphochart we can identify regions in which our computational model produces altered morphologies. Of particular interest to us is the region of non-stabilized mutant structures (shown in green in Fig. 1l and in Fig. 3), as these morphologies represent the potentially invasive mutants of the non-tumorigenic epithelial baseline *MCF10A*. Out of the five different structures that emerged from our simulations, four can be qualitatively matched with morphologies from experimental mutants of *MCF10A* cells. We describe below the morphological similarities between experimental and simulated structures. It must be stressed, however, that further experiments are needed in order to validate or falsify model predictions by comparing model parameter vectors to experimental measurements.

A non-polarized structure (Fig. 3i) showing no cell growth resembles the *MCF10A-HER2* mutant (Fig. 3I) that at day 24 consists of a mass of cells with no detectable staining for proliferation or apoptosis. This simulation was run with low thresholds for both cell growth and cell ECM receptors, and a high threshold for cell death receptors (lower left corner of the mutant parameter space in Fig. 3). This suggests that both cell proliferation and apoptosis were upregulated in comparison to the parental non-tumorigenic cell line, but since the ECM threshold was downregulated all cells became growth-arrested either due to cell-cell adhesion (inner cells) or cell-ECM adhesion (outer cells). A non-stabilized structure with growing cells observed only on the outer rim (Fig. 3ii) is similar to the *MCF10A-HER2-Bcl2* mutant (Fig. 3II) that at day 24 forms a solid cluster of cells with a few outer cells stained with Ki67 nuclear marker showing proliferating events. Matching computational morphologies were obtained when all three thresholds were chosen to be high, such that no cell death was detectable, and all inner cells were in contact inhibition, thus non-growing, but most outer cells have not reached the growth arrested state and therefore continued to proliferate (region ii in the parameter space in Fig. 3). A disorganized acinus with multiple cells growing throughout the cell cluster, and with sporadic apoptotic events (Fig. 3iii) resembles the *MCF10A-HER2-YVMA* mutant (Fig. 3III) that at day 24 forms an irregular mass of cells with numerous proliferative cells located both inside the cluster and on its outer rim. This simulation was run with high thresholds of ECM and apoptotic receptors (both upregulated in comparison to the parental cell line), but a low threshold of growth sensors resulting in frequent proliferations even in the center of the cluster due to diminished contact inhibition response (region iii in Fig. 3). A non-stabilized structure with multiple proliferating and apoptotic events (Fig. 3iv) is similar to the morphology of the *MCF10A-HER2-E7* mutant (Fig. 3IV). In this case the proliferative and apoptotic thresholds were set to a lower level, resulting in numerous growing and dying cells in the simulated acinus. However, the ECM threshold was set high, such that the emerging structures did not reach the growth arrested state by day 24 in culture (right upper corner of the mutant region in Fig. 3). A non-polarized but hollow acinus with an outer rim containing multiple growing cells (Fig. 3v) was simulated by choosing lower values for all three cell receptors thresholds, resulting in a hollow inner lumen with a constantly growing rim of outer cells (right lower corner of the mutant region in Fig. 3). However, to our knowledge there is no experimental data matching this simulated morphology of a *MCF10A* mutant.

These distinct acinar architectures simulated by our model and indicated in Fig. 3 and Fig. S3 arise for different combinations of all three receptor thresholds defining cell sensitivity to proliferative, apoptotic and ECM signals. By mapping various experimental morphologies of *MCF10A* genetic mutants onto the non-stabilized region of the *MCF10A*-tuned morphochart we can estimate the relative changes in the proliferation, death and ECM sensitivities between the mutated and the parental cell lines, *i.e.*, we can indicate whether a certain cellular process is up- or down-regulated in comparison to the parental cell line. These back estimated values effectively link genetic mutations to cell life processes via the generated multicellular morphology and can be used to guide further experimentation.

### *IBCell* morphocharts case study: *MCF10A-HER2-YVMA* mutant

**Figure 4:**
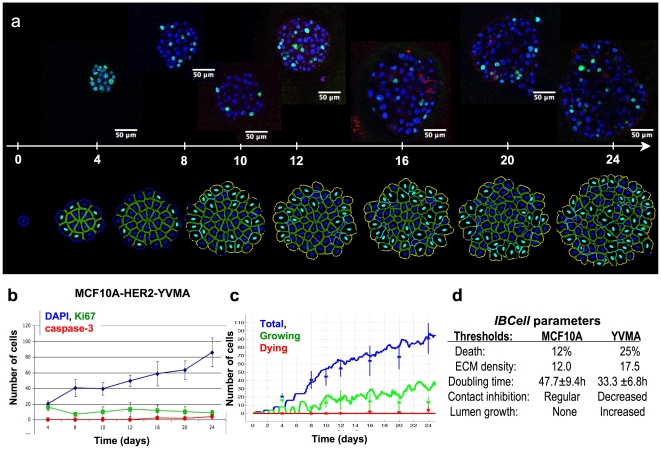
Adjusting the *MCF10A*-tuned model to quantitatively match *MCF10A-HER2-YVMA* data. (**a**) Representative central cross sections from experimental (above the time line) and simulated (below the time line) *MCF10A-HER2-YVMA* mutant at days: 4, 8, 10, 12, 16, 20 and 24, stained for cell nuclei (DAPI, blue), cell proliferation (Ki67, cyan) and cell apoptosis (caspase-3, red). (**b**) Evolution curves of proliferating (green), apoptotic (red) and the total number of cells (blue) collected from experimental data. (**c**) The corresponding evolution curves from computational simulations; stars represent the average value from experimental data with vertical lines showing standard deviations. (**d**) A set of model parameters that differ between simulations reproducing both experimental systems.

To illustrate how the *IBCell* morphocharts can be employed to shed light on phenotypic differences between normal and mutated cells, we used the *MCF10A*-tuned model as a starting point and adjusted its parameters (following the tuning procedure described in the Methods section and the search tree depicted in Fig. 1h) to quantitatively match the experimental data collected from *MCF10A-Her2-YVMA* (called *YVMA* thereafter) cells carrying a constitutively active *HER2* mutant. This allowed us to identify which aspects of the non-tumorigenic baseline needed to be changed in the computational model in order to simulate both the morphology of this specific mutant cell line and its developmental dynamics. Typical cross sections from the *YVMA* experimental data collected over a period of 24 days are shown in the top row of Fig. 4a, whereas the bottom row shows corresponding computational outcomes. The counts of proliferating (green), apoptotic (red) and the total number of cells (blue) from the experimental *YVMAs* (quantified using *BioSig*, see Methods) are shown in Fig. 4b and from the simulated structures in Fig. 4c. The table in Fig. 4d lists the set of model features that were set differently in simulations reproducing the *MCF10A*-like hollow acini and the non-polarized and non-stabilized multicellular clusters typical of *YVMA* mutants.

We assumed that both cell types, *MCF10A* and *YVMA*, have similar diameters of about 20µm. This was estimated from confocal images of the early developmental stages when both cell lines formed solid spheres of cells with no apoptotic events present. The average areas of the central cross sections through *MCF10A* clusters were measured to be 3490±810µm^2^ at day 4, and 7640±1550µm^2^ at day 8 with the average cell counts of 11.5±2 and 25.8±6.3, respectively; whereas the average areas of *YVMA* sections were 6180±1280µm^2^ at day 4 and 11450±2770µm^2^ at day 8 with cell counts of 20.2±4.2 and 40.7±10.7, respectively. In both cases the average cell diameter was about 19.5µm. This clearly highlights that the *YVMA* mutants grow larger and contain more cells than *MCF10A* acini.

To achieve the matched computational results presented in Fig. 4 we first needed to match the number of *YVMA* cells at day 4, which are two times larger than the *MCF10A* samples from the same day. We identified model parameters that directly influence the duration of cell growth leading to the desired increase in cell number. As a result the effective doubling time in our simulations has been changed from 47.7±9.4 hours for *MCF10A* cells to 33.3±6.8 hours for *YVMA* cells. This is consistent with the reported *MCF10A* population doubling time of 48 hours [25], and with the 2- and 4-fold increase in *YVMA* cell number after 4 and 16 days, respectively, when compared to the parental *MCF10A* cells (see [16] as well as Fig. 1g and Fig. 4a). To achieve the desired cell counts and distributions of proliferating cells that match the *YVMA* experimental data at later stages we needed to release the constraints of cell contact inhibition and remove the restriction blocking the inner cells' receptors from sensing free luminal space. We have previously shown that removing these constraints may lead to complete repopulation of the empty lumen [12]. Also, the final morphologies of the *YVMA* mutant are much larger than the *MCF10A* acini, so to reach comparable sizes in our simulations we needed to increase the ECM sensors threshold to prevent or delay acini stabilization. This modification can be interpreted as making the computational *YVMA* cells less sensitive to ECM binding or ECM contact inhibition. Confocal images of *YVMA* clusters show very limited staining for caspase-3 (apoptotic marker) and no lumen formation even at later stages in contrast to *MCF10A* acini that became completely hollow at day 20. In fact, in both experimental systems the quantitative data did not show any significant apoptotic cell death (1–2 cells on average at days 12–20). This may be due to the fact that the apoptotic staining of caspase-3 is detectable only for a few hours and only at the later stages of cell apoptotic death, whereas the experimental data was collected every 4 days. Alternatively, other forms of cell death, such as autophagy [26] or entosis [27] may be responsible for clearing the *MCF10A* lumen. To avoid formation of the luminal space we set the threshold for death sensors significantly higher in the *YVMA* simulations in comparison to *MCF10A* (Fig. 4d), and apoptotic cells were then seen to emerge sporadically (video S2). With these parameters our simulated results for both the total number of cells and the number of growing cells fall within the range of experimental measurements (Fig. 4c), and the distributions of proliferating and apoptotic cells also match the experimental samples (Fig. 4a).

The *MCF10A-HER2-YVMA* tuned simulation of the *IBCell* model led to identification of changes in cell doubling time, lower sensitivity to contact inhibition, modifications in ECM-dependent inhibition of cell proliferation and luminal space promotion of cell growth, that together resulted in the emergence of *YVMA*-like morphologies that quantitatively agreed with our experimental data. These model predictions (Fig. 4d) may be further investigated experimentally in order to confirm or rule out our findings.

## Discussion

In this paper we presented an integrative approach combining *in vitro* experiments, confocal image analysis and quantification, and high throughput simulation studies to understand the relationship between phenotypic and molecular changes in certain mutated cells when compared with their parental non-tumorigenic cell line. We used the previously developed *IBCell* model of epithelial morphogenesis to simulate the formation of a generic acinar structure composed of a shell of tightly packed epithelial cells enclosing the hollow lumen. Subsequently, we tuned this model with quantitative experimental data collected from several samples of *MCF10A* cells grown in three-dimensional cultures. This allowed us to identify starting parameters that served as a seed for constructing a 3D morphochart, *i.e.*, a collection of final morphologies produced by *IBCell* when the model parameters were varied systematically around these baseline values. Next we used this *IBCell* morphochart to identify regions in which our computational model produced structures that quantitatively agreed with *MCF10A* data, as well as those that were morphologically different from the hollow monolayered acini. We then mapped morphologies of four *MCF10A* mutants onto the *IBCell* morphochart to identify the cell phenotypic changes in terms of three cellular processes: proliferation, apoptosis, and ECM-dependent growth inhibition, potentially underlying these mutants. Finally, we examined more closely one specific mutant, *i.e.*, *MCF10A-HER2-YVMA*, and adjusted *MCF10A*-tuned model to quantitatively match the *YVMA* data. This led us to identify the up- and down-regulated cellular processes responsible for observed qualitative and quantitative changes. These computational findings need to be examined experimentally, however, this is beyond the scope of this paper.

It is often a major experimental challenge to acquire certain quantitative data from 3D culture systems, particularly at the cellular scale. Our *IBCell* model has allowed us to acquire a range of quantitative measurements in terms of both individual cellular phenotypes and the whole cluster morphology. Analysis of these measurements revealed that the time of cell growth depends on what fraction of cell surface is exposed to external medium and is not in adhesive contact with other cells. Therefore, the first few cells of the acinus (that have only a few neighbors to adhere to) grow much faster than the cells at the later stages that have a well-developed adhesive neighborhood contributing to their growth arrest. Though the molecular details of contact inhibition are not entirely understood, it is believed that as the number of adherens junctions on the plasma membrane increases, proliferation decreases proportionally. In a 3D setting, contact inhibition is likely more closely related to epithelial polarization, whereby plasma membranes become committed to basolateral and apical domains. Our model recapitulates this mechanism.

The *IBCell* model naturally links multicellular, cellular and molecular scales by allowing us to directly compare mutant and normal cell lines in terms of their phenotypic and morphological changes. Thus enabling a computational investigation of the impact that different cell phenotypes can have on the emerging multicellular structures they produce both as an end point and dynamically as they develop. This may also suggest ways to experimentally investigate the underlying molecular mechanisms.

In the current implementation of *IBCell* we considered cell sensitivity to proliferative and apoptotic signals, cell contact inhibition and ECM-dependent inhibition of cell proliferation. However, other phenotypic characteristics can also be taken into consideration, such as the orientation of cell division, cell motility, response to metabolic factors or to various anti-cancer drugs. *IBCell* can easily integrate multiple cellular traits measured independently in different experimental settings. By using high throughput computational simulations and multidimensional *IBCell*-morphocharts we can map multiple cellular traits to their morphogenetic outcomes and identify combinations of model parameters that define subregions in *IBCell*-morphocharts corresponding to experimentally observed morphologies, and thus determine the common ranges of individually measurable traits for each morphological structure. It is worth to indicate that multiple, differently networked mechanisms, implemented in different ways can give rise to essentially the same phenomena (e.g. multicellular structures of distinct types), thus it is important to validate model findings with wet-lab experiments.

By developing a method that maps mutant morphologies onto simulated ones we have generated a means of linking the morphological and molecular scales via computational modeling. High throughput simulation studies, with the systematically generated model parameter space can point to the altered cell-cell and cell-microenvironment interactions, as well as to changes in cell intrinsic sensitivity to the extrinsic cues. This in turn may guide further experimentation in order to dissect the underlying molecular mechanisms. This procedure of mapping changes in epithelial morphology to cellular phenotypes and to the underlying cancer mutations can also enable quantitative transformations of molecular to pathological findings and *vice versa*. *IBCell* in combination with 3D acini cultures can form a new computational/experimental platform for suggesting the link between histopathology of neoplastic lesions and underlying molecular defects. These computationally mapped values effectively link genetic mutations to cellular traits and can be used to guide further experimentation and to identify relationships between mutations and early cancer tissue lesions.

## Methods

### Experimental methods

A human mammary non-tumorigenic epithelial cell line *MCF10A* and its four mutants expressing *HER2*, *HER2-Bcl2* (HER2 and Bcl2), *HER2-E7* (HER2 and HPV E7), *HER2-YVMA* (HER2 mutant containing a G776^YVMA^ insertion in exon 20), respectively, were grown in 3D cultures by seeding on Growth Factor Reduced Matrigel (BD Biosciences, San Jose, CA) in 8-well chamber slides. The developed multicellular acini were collected every four days for 20 days for the parent cell line *MCF10A* and *MCF10A-HER2-YVMA*, and at end of day 24^th^ for *HER2*, *HER2-Bcl2*, *HER2-E7* and *HER2-YVMA*. All samples were stained with nuclear marker, DAPI, and with antibodies against cleaved caspase-3 and Ki-67. Confocal analyses were performed with an inverted Zeiss LSM-510 confocal microscopy system (Zeiss, Germany). The images of central acinar cross sections were subsequently used to count the numbers of viable (DAPI-positive), proliferating (Ki67-positive) and apoptotic (caspase-3-positive) cells. This analysis was performed using the *BioSig* software (LBNL National Laboratory, Berkeley CA).

### Quantification of experimental samples using *BioSig*

*BioSig* is a bioinformatics framework of integrated image acquisition, annotation, and hierarchical image abstraction to create a database that registers localization and intensity information about multiple targets along with positional references and morphological features [28], [29]. It was originally developed at the Lawrence Berkeley National Laboratory (LBNL) and is accessible through the worldwide web. The first step of this method includes transfer of raw, high-resolution images to the online *BioSig* bioinformatics repository. Using the nuclear marker DAPI as a guide for individual cells present in each acinar structure, images were segmented using a radial voting function [30]. Briefly, this technique includes constraining the solution to provide seeds corresponding to the nuclear regions of all cells, which are calculated based on fluorescence intensity and geometric constraints. Once these seeds are established, Voronoi tessellation provides the local neighborhood where each nucleus resides; this locality is then further partitioned based on its intensity distribution using level sets methods [31]. Additionally, Voronoi tessellation enables quantification of signal within and outside the nuclear regions. As a result, quantification of fluorescence signal (e.g., caspase-3, Ki67) becomes more accurate. These types of analyses are intended to capture inherent heterogeneity in cellular responses and to remove bias associated with user interactions.

### *IBCell* model equations

**Figure 5:**
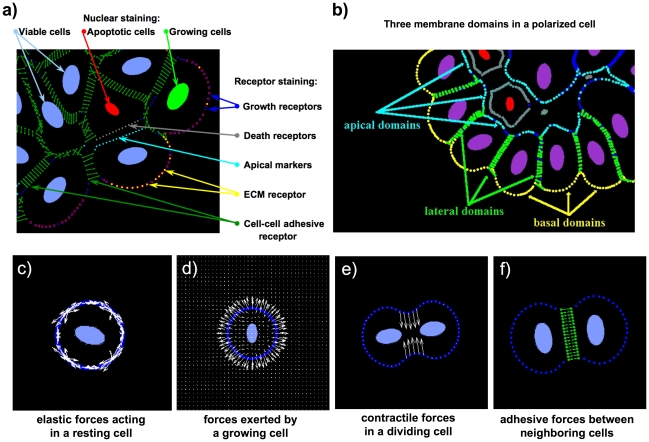
IBCell model computational implementation. (a) Color-coded cell membrane receptors/sensors: growth sensors (blue), death receptors (grey), apical markers (cyan), ECM receptors (yellow), cell-cell adhesive receptors (green); Color-coded nuclear staining: nuclei of growing cells (green), nuclei of apoptotic cells (red), nuclei of viable cells (blue). (b) Color-coded representation of epithelially polarized cells containing three membrane domains: basal consisting of ECM receptors (yellow), lateral consisting of cell-cell adhesive receptors (green) and apical consisting of apical markers (cyan). (c) Forces exerted by an elastic cell membrane modeled as a collection of short liner springs. (d) Forces exerted by an expanding (growing) cell. (e) Contractile forces exerted by a dividing cell. (f) Adhesive forces exerted by two neighboring nearby cells.

The *IBCell* model is based on the Immersed Boundary Method [32], a fluid-dynamics framework suitable to model interactions between deformable elastic bodies (such as eukaryotic cells) and the viscous incompressible fluid (such as cell cytoplasm or the extracellular matrix). The cell structure consists of an elastic plasma membrane modeled as a network of linear springs that defines cell shape and encloses the fluid providing cell mass (Fig. 5c). These individual cells can interact with other cells and with the environment via a set of discrete membrane receptors/sensors located on the cell boundary (Fig. 5a). These sensors can be engaged in adhesion either with one of the neighboring cells or with the extracellular matrix, or can be used to sense the presence of other cells or the ECM in cell local vicinity. The host cell can initiate certain cell life processes, such as proliferation, division, apoptotic death or epithelial polarization, based on its membrane receptors/sensors configuration (a distribution of growth, death, apical, cell-cell and cell-ECM adhesion sensors, Fig. 5a). More precisely, cell growth is modeled by placing point sources and sinks around the cell boundary to model transport of fluid through the cell membrane (Fig. 5d), and once the cell area is doubled the contractile ring is formed by introducing springs between opposite points on the cell boundary that upon contraction split the cell into two daughters (Fig. 5e). Cell-cell adhesion is modelled by introducing a short liner spring between adhesive receptors on two neighboring cells (Fig. 5f). Cell epithelial polarity is acquired by developing three distinct membrane domains: basal, defined by cell membrane sensors contacting the external media; lateral, defined by cell sensors being in contact with other cells; and apical, facing the hollow lumen (Fig. 5b). Cell apoptotic death is modelled by placing point sinks and sources along the membrane of the whole cell to release fluid from the cell interior to the extracellular space. The *IBCell* model is fully deterministic (*i.e.*, given the same starting parameters, such as numbers of cell membrane sensors and values of receptor thresholds), the model will produce the same morphological output. The *IBCell* model is governed by the following set of equations.
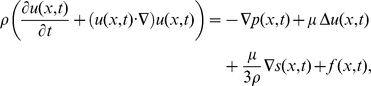
(1)

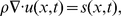
(2)

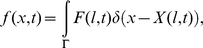
(3)

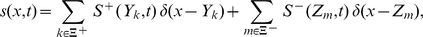
(4)

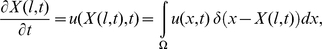
(5)

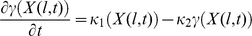
(6)


In this system, Eq. (1) is the Navier-Stokes equation of a viscous incompressible fluid defined on the Cartesian grid 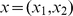, where 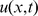 is the fluid velocity, 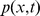 is the fluid pressure, 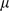 is the fluid viscosity, 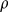 is the fluid density, 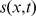 is the local fluid expansion, and 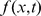 is the external force density. Eq. (2) is the law of mass balance. Interactions between the fluid and the material points 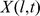 on cell boundaries 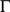 (where 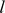 is a material point index) and at point sources 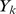 and sinks 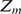 placed in the cell local microenvironment are defined in Eqs. (3)–(5). Here, the force density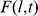 defined on cell boundaries, and the sources 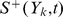 and sinks 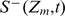 defined in the cell microenvironment are applied to the fluid using the two dimensional Dirac delta function 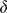, while all material boundary points 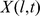 are carried along with the fluid. The boundary forces 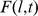 arise from elastic properties of cell boundaries, from cell-cell adhesion and from contractile forces splitting a cell during its division. The sources 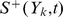 and sinks 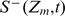 are chosen such that they balance around each cell separately. The kinetics of ECM proteins 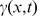 is defined along the cell boundaries and includes: constant secretion (at a rate 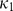) along the cells' basal domains and its decay (at a rate 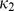) around all cells' boundaries. More details about model implementation and computational complexity can be found in [2], [11]. Overall, each simulation reproducing a single acinar morphology takes about 20–40 hours of computational time on a standard single processor Mac Pro desktop computer.

### IBCell model tuning with experimental data

The *IBCell* tuning process is based on fitting the simulated acinar structures and cell counts to experimental data and measurements collected at certain time points. This is done by constructing the search tree (Fig. 1h and Fig.S1) that discriminates between these model parameters that can generate the desired structure and cell counts at the consecutive time points, and those that lead to false morphologies. For example, in order to attain the desired number of cells at day 4, one of the following parameters may be modified: i) cell-cell adhesion may be reduced resulting in the increased number of growth receptors and subsequently in larger amounts of water pumped into the host cell; thus the total cell area will be doubled faster and the total number of cells will increase faster; ii) cell maturation time (*i.e.*, time used by the host cell to rest after the mitotic division is completed) may be shortened; iii) cell doubling time may be reduced by choosing a larger fluid source strength for all growing cells; iv) the growth receptor threshold may be reduced that will require a smaller percentage of cell membrane receptors to be acuired to trigger the process of cell growth (for more details on implementation of cell life processes in *IBCell* see [2], [11]). The simulated results are then compared qualitatively and quantitatively to experimental data and these branches of the search tree which do not match with experiments are neglected in further analysis. In the case presented in Fig. 1h both peripheral branches (at the top and bottom of the search tree) will be cut as the number of cells in both branches is significantly different at day 4 than in the experimental data (compare Fig. 1a day 4, and Fig. 1h). It is worth noting that in the initial phase the most significant model parameters are those that result in comparable cell proliferation, whereas the parameters influencing cell apoptosis (f.e. the threshold for death sensors, time delay needed for cell cytoskeleton to collapse, time delay in cell-cell adhesion disassembly), cell polarization and acinar stabilization (f.e. a threshold for cell-ECM adhesion, distance for cell-cell adhesion links assembly and disassembly, time dalay for the apical sensors emergence) are more important in matching the simulated and experimental data in the later stages of acinar development. A more detailed exapmle of constructing the parameter tree including the matching and false morphologies is presented in Figure S1.

## Supporting Information

Figure S1 A scheme of model tuning. A scheme illustrating the process of model tuning with experimental data collected at the particular time points. A search parameters tree allows for discrimination between parameters showing promise to generate the desired structures and those that lead to false morphologies. The simulated results are systematically compared, qualitatively and quantitatively, to experimental data in order to prune (dashed arrow lines) these branches of the search tree that do not match. The simulation process starts with a single cell (Day 0 figure a), and several model parameters are selected in order to attain the desired number of cells and the multicellular structure at day 4. These branches of the search tree which do not match with experiments (*i.e.*, both peripherial branches in figures a and d, Day 4) are neglected in further analysis. After fixing parameters for which the simulated acini agree with experimental ones, another parameters are inspected, and the simulations are run again from a single cell in order to compare the simulated results with the next time point. The search-and-simulation process is repeated until all time points are fitted. It is is worth noting that in the initial phase the most significant are these model parameters that result in comparable cell proliferation (f.e., strength of cell-cell adhesion, length of cell maturation time, strength of fluid sources, a threshold for growth receptors), whereas the parameters influencing cell apoptosis (f.e. a threshold for death sensors, time delay needed for cell cytoskeleton to collapse, time delay in cell-cell adhesion disassembly), cell polarization and acinar stabilization (f.e. a threshold for cell-ECM adhesion, distance for cell-cell adhesion links assembly and disassembly, time delay for the apical sensors emergence) are more important in matching the simulated and experimental data in the later stages of acinar development. In the presented example, only two paths (*a-b-b-b-…-a*, and *a-b-c-c-…-b* at days 0, 4, 8, 12 and 20, respectively) lead to acinar morphologies comparable with experimental data (see also Fig. 2i and Fig. 2ii). All other paths are neglected since the computationally generated morphologies and cell counts do not match the experimentally collected data.
(2.11 MB TIF)

Figure S2 A developmental sequence of five acinar morphologies. A sequence of consecutive stages in the development of five distinct acinar morphologies at days a) 0, b) 6, c) 12, d) 18, e) 24. The final acinar structures correspond to those shown in Fig. 2. Acinus i) and Acinus ii) correspond to structures that qualitatively and quantitatively agree with experimental data acquired from *MCF10A* cells. Three other structures have similar cell counts at day 6 (b), but they either grow too large (Acinus iii) or too small (Acinus iv and Acinus v). The five simulations differ only in three receptor thresholds for cell growth (G), death (D) and ECM (E) receptors emergence. The corresponding parameter vectors V = (G,D,E) are: Acinus i) V = (25%,10%,14.5), Acinus ii) V = (15%,12.5%,12), Acinus iii) V = (15%,12.5%,14.5), Acinus iv) V = (20%,17.5%,9.5), Acinus v) V = (15%,17.5%,7). Color-coded cell membrane receptors/sensors include: growth sensors (blue), death receptors (grey), apical markers (cyan), ECM receptors (yellow), cell-cell adhesive receptors (green). Color-coded nuclear staining includes: nuclei of viable cells (blue), nuclei of apoptotic cells (red).
(2.71 MB TIF)

Figure S3 A developmental sequence of five mutant morphologies. A sequence of consecutive stages in the development of five distinct mutant morphologies at days a) 0, b) 6, c) 12, d) 18, e) 24. The final mutant morphologies correspond to those shown in Fig. 3. The five simulations differ only in three receptor thresholds for cell growth (G), death (D) and ECM (E) receptors emergence. The corresponding parameter vectors V = (G,D,E) are: Mutant i) V = (0%,40%,12.5), Mutant ii) V = (5%,45%,12.5), Mutant iii) V = (0%,65%,12.5), Mutant iv) V = (10%,5%,12.5) and lumen promotion growth, Mutant v) V = (0%,5%,12.5). Intra- and intercellular elements are color-coded as in Fig. S2.
(2.68 MB TIF)

Video S1 Development of an epithelial acinus. Development of an epithelial acinus tuned with *MCF10A* experimental data over the time corresponding to 20 days in culture. Left panel shows a developing morphology of the *MCF10A* acinus. Right panel shows the evolution of cell count curves matching the experimental data represented as stars with vertical bars denoting the standard deviation.
(0.47 MB AVI)

Video S2 Development of an epithelial acinar mutant. Development of an epithelial acinar mutant tuned with *MCF10A-HER2-YVMA* experimental data over the time corresponding to 24 days in culture. Left panel shows a developing morphology of the mutant. Right panel shows the evolution of cell count curves matching the experimental data represented as stars with vertical bars denoting the standard deviation.
(4.58 MB AVI)
