# Supplementary figures and images for: Linking Changes in Epithelial Morphogenesis to Cancer Mutations Using Computational Modeling
Source: PLoS Comput Biol. 2010 Aug 26;6(8):e1000900. doi: 10.1371/journal.pcbi.1000900 (PMC2928778; doi:10.1371/journal.pcbi.1000900)

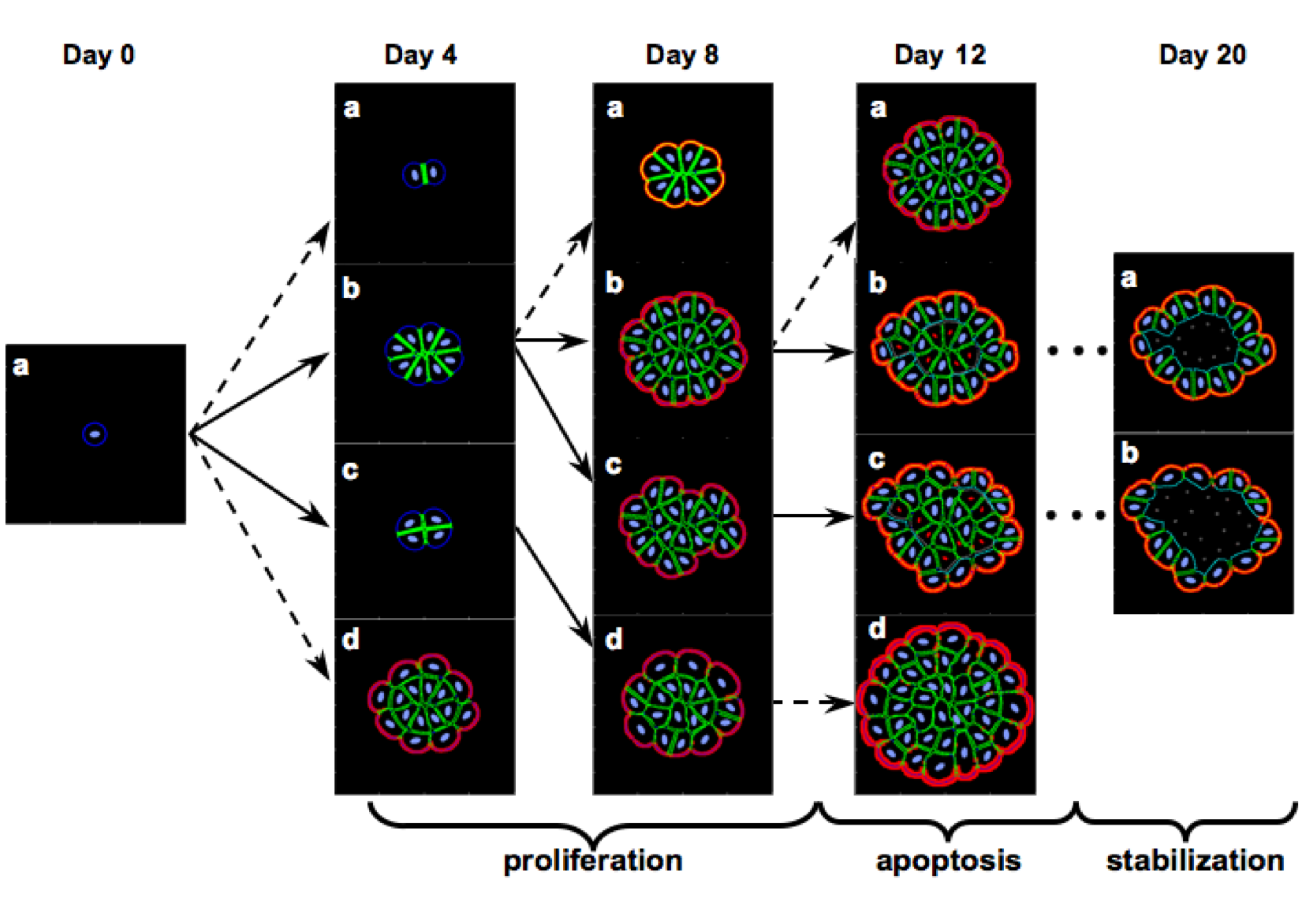

Supplement: Figure S1 — A scheme of model tuning. A scheme illustrating the process of model tuning with experimental data collected at the particular time points. A search parameters tree allows for discrimination between parameters showing promise to generate the desired structures and those that lead to false morphologies. The simulated results are systematically compared, qualitatively and quantitatively, to experimental data in order to prune (dashed arrow lines) these branches of the search tree that do not match. The simulation process starts with a single cell (Day 0 figure a), and several model parameters are selected in order to attain the desired number of cells and the multicellular structure at day 4. These branches of the search tree which do not match with experiments (i.e., both peripherial branches in figures a and d, Day 4) are neglected in further analysis. After fixing parameters for which the simulated acini agree with experimental ones, another parameters are inspected, and the simulations are run again from a single cell in order to compare the simulated results with the next time point. The search-and-simulation process is repeated until all time points are fitted. It is is worth noting that in the initial phase the most significant are these model parameters that result in comparable cell proliferation (f.e., strength of cell-cell adhesion, length of cell maturation time, strength of fluid sources, a threshold for growth receptors), whereas the parameters influencing cell apoptosis (f.e. a threshold for death sensors, time delay needed for cell cytoskeleton to collapse, time delay in cell-cell adhesion disassembly), cell polarization and acinar stabilization (f.e. a threshold for cell-ECM adhesion, distance for cell-cell adhesion links assembly and disassembly, time delay for the apical sensors emergence) are more important in matching the simulated and experimental data in the later stages of acinar development. In the presented example, only two paths (a-b-b-b-…- [file pcbi.1000900.s001.tif]

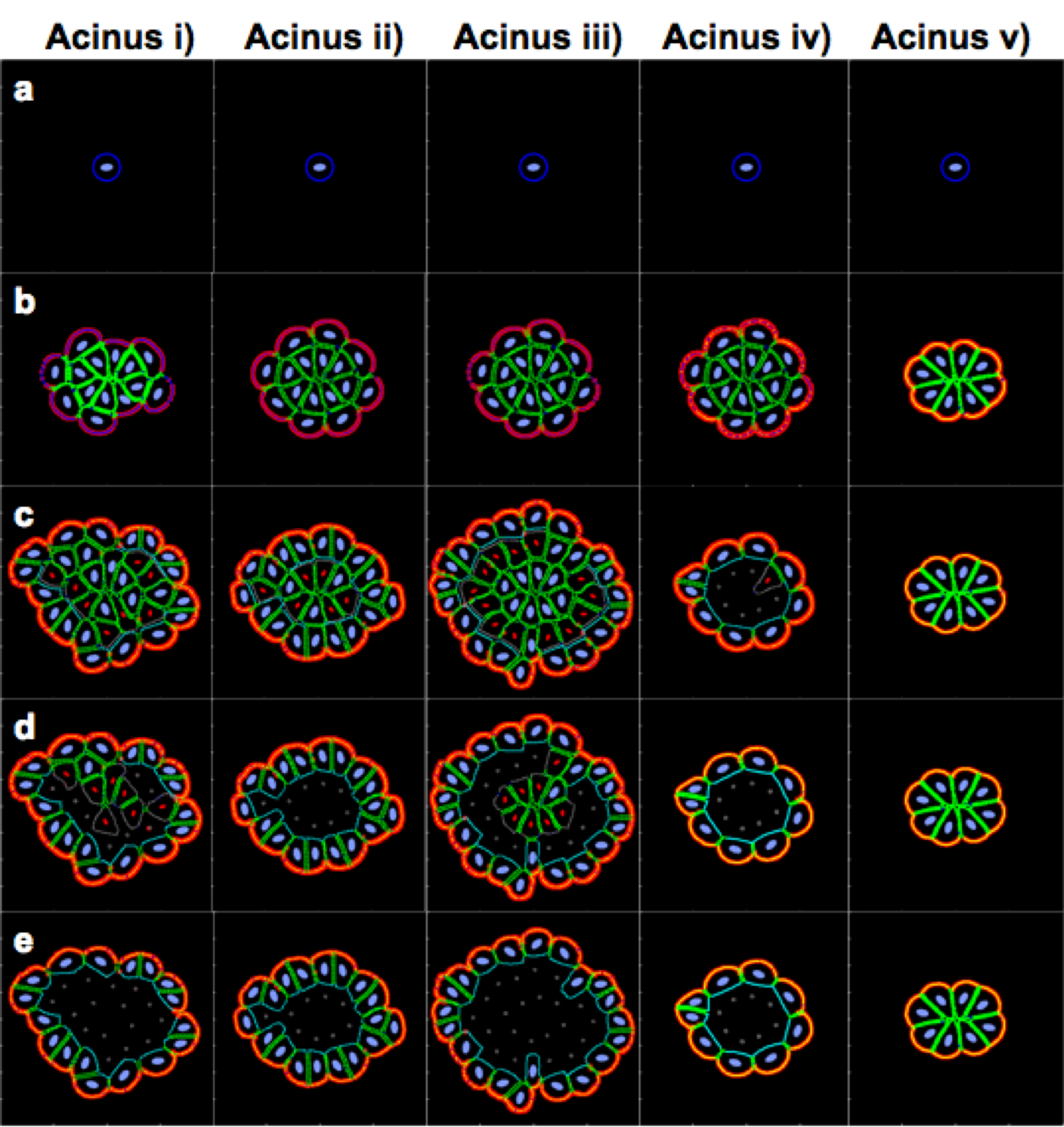

Supplement: Figure S2 — A developmental sequence of five acinar morphologies. A sequence of consecutive stages in the development of five distinct acinar morphologies at days a) 0, b) 6, c) 12, d) 18, e) 24. The final acinar structures correspond to those shown in Fig. 2. Acinus i) and Acinus ii) correspond to structures that qualitatively and quantitatively agree with experimental data acquired from MCF10A cells. Three other structures have similar cell counts at day 6 (b), but they either grow too large (Acinus iii) or too small (Acinus iv and Acinus v). The five simulations differ only in three receptor thresholds for cell growth (G), death (D) and ECM (E) receptors emergence. The corresponding parameter vectors V = (G,D,E) are: Acinus i) V = (25%,10%,14.5), Acinus ii) V = (15%,12.5%,12), Acinus iii) V = (15%,12.5%,14.5), Acinus iv) V = (20%,17.5%,9.5), Acinus v) V = (15%,17.5%,7). Color-coded cell membrane receptors/sensors include: growth sensors (blue), death receptors (grey), apical markers (cyan), ECM receptors (yellow), cell-cell adhesive receptors (green). Color-coded nuclear staining includes: nuclei of viable cells (blue), nuclei of apoptotic cells (red). (2.71 MB TIF) [file pcbi.1000900.s002.tif]

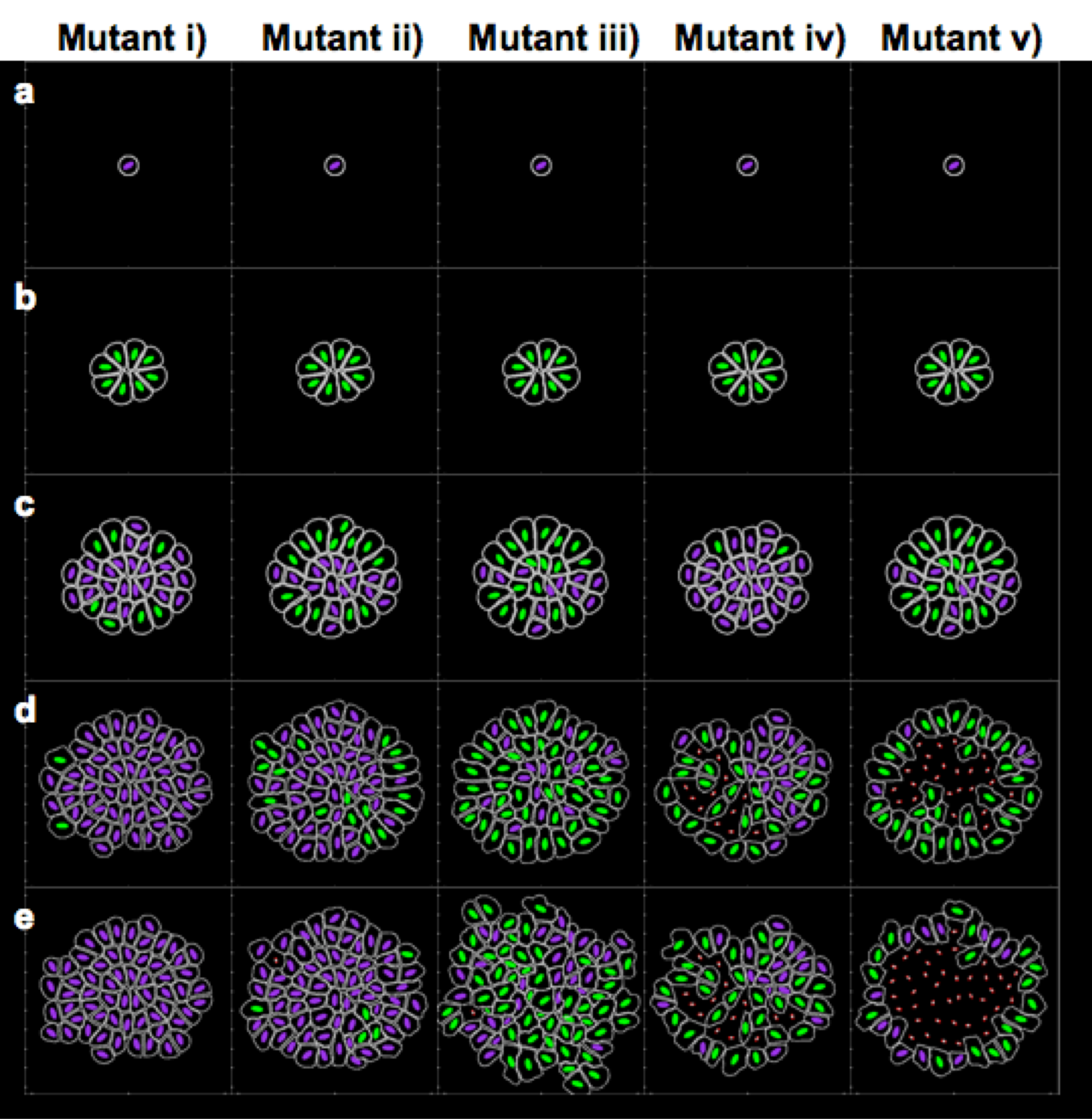

Supplement: Figure S3 — A developmental sequence of five mutant morphologies. A sequence of consecutive stages in the development of five distinct mutant morphologies at days a) 0, b) 6, c) 12, d) 18, e) 24. The final mutant morphologies correspond to those shown in Fig. 3. The five simulations differ only in three receptor thresholds for cell growth (G), death (D) and ECM (E) receptors emergence. The corresponding parameter vectors V = (G,D,E) are: Mutant i) V = (0%,40%,12.5), Mutant ii) V = (5%,45%,12.5), Mutant iii) V = (0%,65%,12.5), Mutant iv) V = (10%,5%,12.5) and lumen promotion growth, Mutant v) V = (0%,5%,12.5). Intra- and intercellular elements are color-coded as in Fig. S2. (2.68 MB TIF) [file pcbi.1000900.s003.tif]
